# Molecular characterization and phylogenetic analysis of a dengue virus serotype 3 isolated from a Chinese traveler returned from Laos

**DOI:** 10.1186/s12985-018-1016-5

**Published:** 2018-07-24

**Authors:** Ling Mo, Jiandong Shi, Xiaofang Guo, Zhaoping Zeng, Ningzhu Hu, Jing Sun, Meini Wu, Hongning Zhou, Yunzhang Hu

**Affiliations:** 1 Institute of Medical Biology, Chinese Academy of Medical Sciences and Peking Union Medical College, Kunming, 650118 China; 2 Yunnan Key Laboratory of Vaccine Research and Development of Severe Infectious Disease, Kunming, 650118 China; 3 Yunnan Provincial Center of Arborvirus Research, Yunnan Provincial Key Laboratory of Vector-borne Diseases Control and Research, Yunnan Institute of Parasitic Diseases, Pu’er, 665000 Yunnan China

**Keywords:** Dengue virus serotype 3, Molecular characterization, Phylogenetic analysis, Genotype

## Abstract

**Background:**

Dengue virus (DENV) infection caused by international visitors has become a public health concern in China. Although sporadic imported cases of DENV have been documented in Yunnan, China since 2000, a complete genome sequence of dengue virus serotype 3 (DENV-3) imported from Laos is still not available. Here, we report the first complete genome sequence and genomic characterization of a DENV-3 strain (YNPE3) isolated from a patient returned from Laos.

**Methods:**

Viral isolation from the patient’s serum was performed using mosquitoes C6/36 cells. Reverse transcriptase polymerase chain reaction (RT-PCR) was used for identification and serotyping of the virus. The complete sequence was determined by Sanger dideoxy sequencing. Homology analysis was implemented by NCBI-BLAST. Multiple sequence alignment was performed using MegAlign module of the Lasergene 7 software package DNASTAR. MFOLD software was used to predict the RNA secondary structure of 5′ untranslated region (UTR) and 3′ UTR. Phylogenetic analysis, which was based on envelope gene and complete coding sequence, was performed by Maximum-Likelihood method.

**Results:**

RT-PCR analysis confirmed that the virus belonged to dengue virus serotype 3, which was named YNPE3 strain. The full-length genome of the YNPE3 strain was 10,627 nucleotides (nts) with an open reading frame (ORF) encoding 3390 amino acids. Strain YNPE3 shared 98.6–98.8% nucleotide identity with the closely related strains isolated in India (JQ922556, KU216209, KU216208). We observed the deletion of about 40 nts in the 5′ UTR and 3′ UTR of strain YNPE3, and 11 nts (ACGCAGGAAGT) insertion that was present in the 3′ UTR of YNPE3. Compared with prototype strain H87, abundant amino acid substitutions in the YNPE3 strain were observed. Phylogenetic analysis revealed that the YNPE3 strain belonged to genotype III of DENV-3, and that it might be closely related with genotype III strains isolated in Laos and India.

**Conclusions:**

This is the first report of the complete genome sequence and molecular characterization of a DENV-3 isolate imported from Laos. The presented results can further promote disease surveillance, and epidemiological and evolutionary studies of the DENV-3 in Yunnan province of China.

**Electronic supplementary material:**

The online version of this article (10.1186/s12985-018-1016-5) contains supplementary material, which is available to authorized users.

## Background

Dengue infection is a rapidly re-emerging mosquito-borne infectious disease caused by dengue virus (DENV), that affects approximately 390 million people annually [1]. More than 100 countries, spanning from the Americas, the Western Pacific, South-East Asia, and Africa to Europe, have reported severe epidemics of dengue. In fact, more than 3.9 billion people worldwide are estimated to be at transmission risk [2]. Dengue infections cause varying degrees of dengue disease, with major clinical manifestations ranging from asymptomatic Dengue Fever (DF) to serious Dengue hemorrhagic fever (DHF) and Dengue shock syndrome (DSS) [3]. Unfortunately, approximately 50–100 million DF cases and hundreds of thousands of DHF cases occur annually [4, 5]. Since there are still no licensed vaccines or specific antiviral drugs, and since each patient with dengue hemorrhagic fever needs to face exorbitant medical costs, dengue infections have become a serious global health problem, and a heavy socioeconomic burden affecting healthcare systems worldwide.

As the etiologic agent of dengue fever, DENV belongs to the genus *Flavivirus* and family *Flaviviridae* [6, 7] primarily transmitted by *Aedes aegyptis* and *Aedes albopictus* [8, 9], that are known to circulate in the tropical and subtropical regions of the world [8]. DENV is an enveloped, positive-sense, single-stranded RNA virus. The genome of DENV is approximately 11 kb in length, with one open reading frame (ORF) flanked by 5′ and 3′ non-coding regions. A polyprotein is encoded by the ORF, which is cleaved into three structural proteins: C: Capsid glycoprotein; M: membrane glycoprotein; E: envelope glycoprotein; and seven non-structural proteins (NS): NS1, NS2A, NS2B, NS3, NS4A, NS4B and NS5 [10]. Furthermore, DENV is divided into four serotypes(DENV- 1 to DENV-4)based on antigen cross-reactivity. Since there is no cross protection among serotypes, secondary infection of heterologous DENV serotypes often results in severe disease [11]. In addition, each serotype is further divided into distinct genotypes based on viral genome sequencing and evolutionary analysis [12].

Over the past decade, dengue infection has expanded over the vast geographical range [13–16], This may be caused by more frequent international travel, climate change, virus evolution, unplanned urbanization, globalization, population mobility and failure of Aedes mosquitoes control [13, 14, 17, 18]. In Mainland China, the first recorded DENV infection occurred in Guangdong in 1978 [19]. Over the past 30 years, DENV has spread throughout the country. Although the four serotypes have been circulating in Guangdong, Guangxi, Hainan, Fujian, Zhejiang and Yunnan provinces of China [20, 21], the DENV-1 has been a predominate serotype since 1990s [21]. Geographically, provinces such as Guangdong, Guangxi, Hainan, Fujian, and Zhejiang are situated in the southeast coast of China, whereas Yunnan borders with Southeast Asian countries, such as Myanmar, Laos, Thailand, Vietnam which are all considered endemic risk areas [21–25]. The spread of DENV by tourists returning from Southeast countries is common in China, especially in Yunnan Province. Since 2000, most of the DF documented in Yunnan have been sporadic imported cases. In 2013, a large-scale DENV-3 outbreak comprising 1287 indigenous cases and 44 imported cases occurred in Xishuangbanna, Jinghong, Yunnan [26]. Epidemiological analysis showed that this outbreak was closely related with the imported dengue cases from Laos and Myanmar [26, 27], which suggested that imported dengue cases were mainly responsible for outbreaks and the circulation of DENV-3 in Yunnan, China. Due to the special geographical location, humid climate, booming international tourism, population mobility and the spread of the Aedes mosquitoes, Yunnan is at risk of becoming an endemic risk area in the future.

Although dengue cases imported from Laos have been reported in previous studies, they mainly focused on the envelope (E) gene of the imported DENV-3. The complete genome sequence and molecular characterization of the DENV-3 strain imported from Laos are still not available. In the current research, we reported the complete genome sequence and genomic characterization of a DENV-3 strain imported from Laos. The results from this study provide valuable clues for disease surveillance, transmission control and vaccine design.

## Methods

### Source of virus

A 10 year old student, who resides in Pu’er, Yunnan Province, China, went on a 7 day trip to Laos from August 10th to August 16th, 2013. She experienced slight fever during the 4th day of the trip, and influenza like symptoms on the third day after returning home. In August 20th, 2013, clinical diagnosis showed a positive result for NS1 antigen of patient’s serum based on the viral NS1 antigen colloidal gold test using the Dengue Ag Rapid Test (CTK Biotech, Inc., San Diego, CA), in the Jinghong People’s Hospital in China. The medical history of the patient was unremarkable with dengue fever and other flaviviruses disease. Written informed consent was obtained from this patient and her legal guardian. A serum sample was collected from the patient within 3–7 days after the onset of the illness and stored in −80 °C until use.

### Virus isolation and serotyping

The patient’s serum was inoculated into the C6/36 *Aedes albopictus* cell line in RPMI 1640 medium (Biological Industries, USA) supplemented with 2% fetal bovine serum (Biological Industries, USA) at 28 °C in 5% CO2. When 70% cytopathic effect (CPE) was observed, the virus was passaged twice more to prepare viral stocks. Supernatant from infected cells was harvested and stored at −80 °C until used. Viral RNA was extracted from 500 μL of infected culture supernatant by Trizol reagent (Tiangen, Beijing, China) according to the manufacturer’s instructions. For virus identification, reverse transcriptase polymerase chain reaction (RT-PCR) amplification was carried out as previously described [28]. Briefly, viral RNA was reverse transcribed in a BioRad C1000 Cycler system using GoScript™ Reverse Transcription System (Promega, WI, USA) according to the manufacturer’s instructions. The cDNA was used for PCR analysis. Five pairs of primers, which were designated as universal primers D1 and D2 of DENV, and four typing primers D1 and TS1 of DENV-1, D1 and TS2 of DENV-2, D1 and TS3 of DENV-3, D1 and TS4 of DENV-4 (primers were described in Additional file 1: Table S1), were employed to amplify the partial capsid/membrane glycoprotein precursor (PrM) gene of DENV. PCR amplification was conducted with the following program: 2 min at 94 °C, followed by 35 cycles at 94 °C for 30 s, 55 °C for 30 s, 72 °C for 1 min, and a final extension step at 72 °C for 10 min. The specific PCR amplicons were further confirmed by agarose gel electrophoresis. PCR products were visualized by a biospectrum® 815 imaging system (UVP, USA).

### Genome amplification and sequencing

A total of nine overlapping sub-genomic fragments that spanned the complete genomic region were amplified using nine pairs of primers (Additional file 2: Table S2) [29] by RT-PCR. First, viral RNA was reverse transcribed using the GoScript Reverse Transcription System (Promega, WI, USA) following the manufacturer’s instructions. The RT reaction was conducted under the following conditions: 25 °C for 5 min, followed by 1 h at 42 °C, then 72 °C for 15 min. Next, the PCR was carried out in a reaction mixture of 50 μL containing 5 μL of 10 × La Taq Buffer (Mg^2+^ Plus), 8 μL of dNTP mixture (2.5 mM), 2 μM of forward and backward primers, and one unit of high fidelity La Taq DNA polymerase (TaKaRa, Dalian, China). PCR amplification was performed under the following parameters [29]: 94 °C for 5 min, followed by 35 cycles of denaturation at 94 °C for 30 s, annealing at 55 °C for 30 s, extension at 72 °C for 2 min and final extension at 72 °C for 5 min. The PCR results were analyzed by 1% agarose gel electrophoresis and visualized by a biospectrum® 815 imaging system (UVP, USA). Finally, the PCR products were used as templates for bi-directional DNA sequencing by the Sanger dideoxy sequencing method (Invitrogen Ltd., Shanghai).

### Genomic characterization and sequencing analysis

The sequences of nine sub-genomic fragments were assembled into a complete genome by removing overlapping sequences with the DNAMAN software program (v. 8.0.8.789) (Lynnon Biosoft, USA). The assembled viral genome was further aligned by NCBI-BLAST (https://blast.ncbi.nlm.nih.gov/Blast.cgi) to determine the similarity with other virus strains. Multiple sequences alignment analyses of nucleotides and amino acids for six closely related DENV-3 isolates from China, Laos and other countries were performed based on MegAlign module of Lasergene 7 software package DNASTAR (DNASTAR Inc., USA). Further, the secondary structure of 3′ UTR and 5′ UTR of the YNPE3 strain and prototype strain H87 (M93130) of DENV-3 were determined by the MFOLD software package (http://unafold.rna.albany.edu/?q=mfold) [30] using default folding parameters.

### Phylogenetic analysis

Two phylogenetic trees were drawn based on the complete coding sequence (CDS) and E gene of DENV-3. For the E gene, a total of 76 representative strains isolated from different geographical regions in different years were retrieved from GenBank (Additional file 3: Table S3). For the CDS, a total of 75 representative DENV-3 isolates were prepared (Additional file 4: Table S4), the two sets of strains were different with a 10% overlap. The evolutionary history was inferred using the Maximum-Likelihood method [31] of MEGA software (version 7) [32], with a bootstrap test (1000 replicates) [33]. The evolutionary distance was calculated using the Tamura-Nei model [34]. The mutation rate at each site was modeled with a gamma distribution (shape parameter =5). DENV-1 strain Hawaii, DENV-2 strain New Guinea C and DENV-4 strain H241 served as outgroups.

## Results

### Identification and serotyping of virus

**Fig. 1:**
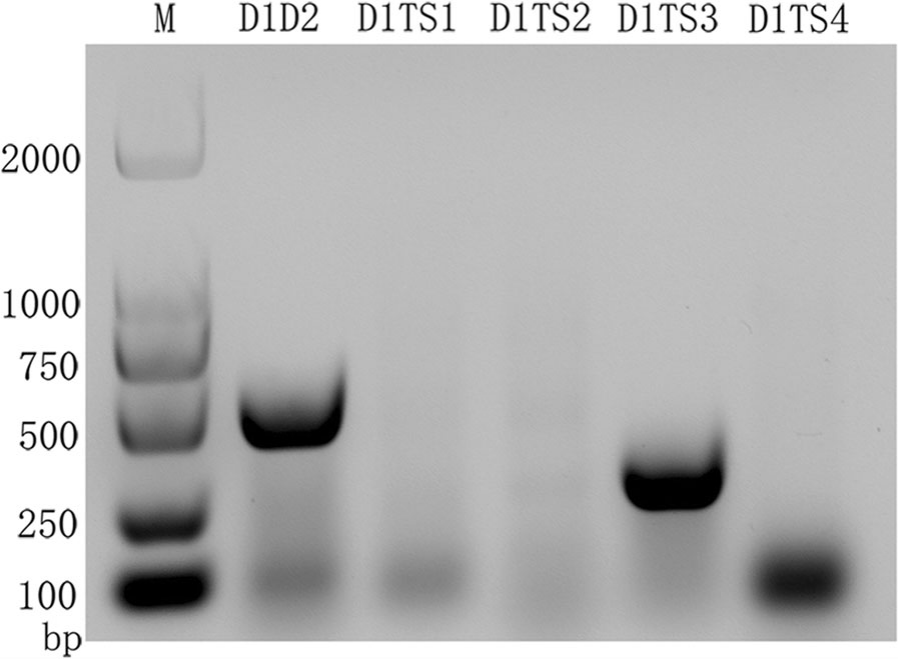
Electrophoresis of typical primer identification of DENV-3. The symbol M indicates DL 2000 DNA marker; The D1D2 indicates the universal amplicon that amplified the partial capsid/premembrane gene (511 bp) of DENV by upstream primer D1 and downstream primer D2. The D1TS1 (482 bp), D1TS2 (119 bp), D1TS3 (290 bp) and D1TS4 (392 bp) are specific amplicons of DENV-1, DENV-2, DENV-3 and DENV-4, respectively

In order to isolate the virus, the patient’s serum was inoculated in cells of the C6/36 cell line until the occurrence of typical CPE, followed by passage twice to increase virus stocks. Compared with normal cells, a typical CPE with the fusion, shedding, aggregation, breaking and suspension were observed in infected cells after 9 days of infection. Furthermore, two fragments with 511 and 290 bp were amplified specifically by RT-PCR with universal primes for DENV and typing primes for DENV-3 respectively, and no specific amplification appeared in the D1TS1, D1TS2 and D1TS4, indicating that the isolate belonged to the serotype 3 of DENV (Fig. 1).

### Complete genome sequence and genomic characterization of YNPE3

**Fig. 2:**
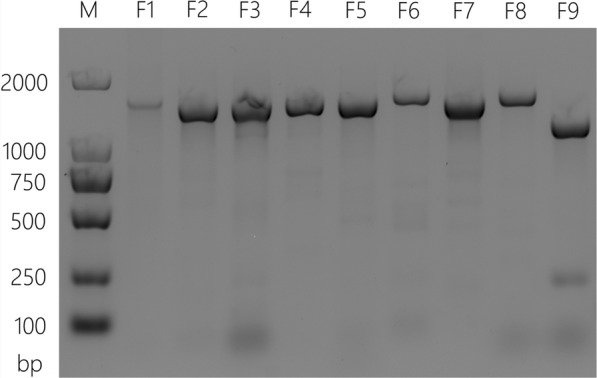
Electrophoresis of the whole genome sequence amplification from YNPE3 strain on a 1% agarose gel. The symbol M indicates DL 2000 DNA marker; and then from left to right the primer sets are: F1:3F1/3R1, F2: 3F2/3R2, F3: 3F3/3R3, F4: 3F4/3R4, F5: 3F5/3R5, F6: 3F6/3R6, F7: 3F7/3R7, F8: 3F8/3R8, F9: 3F9/3R9

To amplify the complete genome sequence of the YNPE3 strain, nine sets of primers were designed to amplify nine overlapping fragments across the whole viral genome. As shown in Fig. 2, the nine overlapping fragments were amplified specifically by RT-PCR. Moreover, the sequences of all the overlapping amplicons assembly revealed a full-length genome with 10,627 nts for the YNPE3 strain. The complete genome sequence was submitted to the GenBank database under the accession number MF370226.1. The YNPE3 strain contained a single ORF that encoded 3390 amino acids (aa) flanked by 5′ UTR (55 nts) and 3′ UTR (399 nts). The nucleotide acid composition of the YNPE3 strain was 32.11% A, 26.03% G, 21.28% T and 20.58% C. The percentage of purine (58.14%) was higher than that of pyrimidine (41.86%).

Further similarity analysis by BLAST showed that the YNPE3 strain had the highest identity with the SG(EHI)D3/15095Y15 strain (KY921907) isolated from Singapore in 2015, it possessed 99.4% nucleotide identity and 99.6% amino acid identity. Followed by three strains from Indian (JQ922556, KU216209, KU216208), they showed to share 98.6–98.8% nucleotide identity with YNPE3 strain. Compared with the prototype strain H87, there was 94.0% nucleotide identity and 97.8% amino acid identity. The strains from Laos (KY849769, KY849770, KY849771, KY849772, KY849773, KY849774 and KY849775) shared 93.1% nucleotide identity and 97.8–97.9% amino acid identity with YNPE3 strain. Yet, the strains from China YN01 (KF824902) and YN02 (KF824903) isolated from Yunnan in 2013, shared 92.9% nucleotide identity and 97.7–97.8% amino acid identity.

**Fig. 3:**
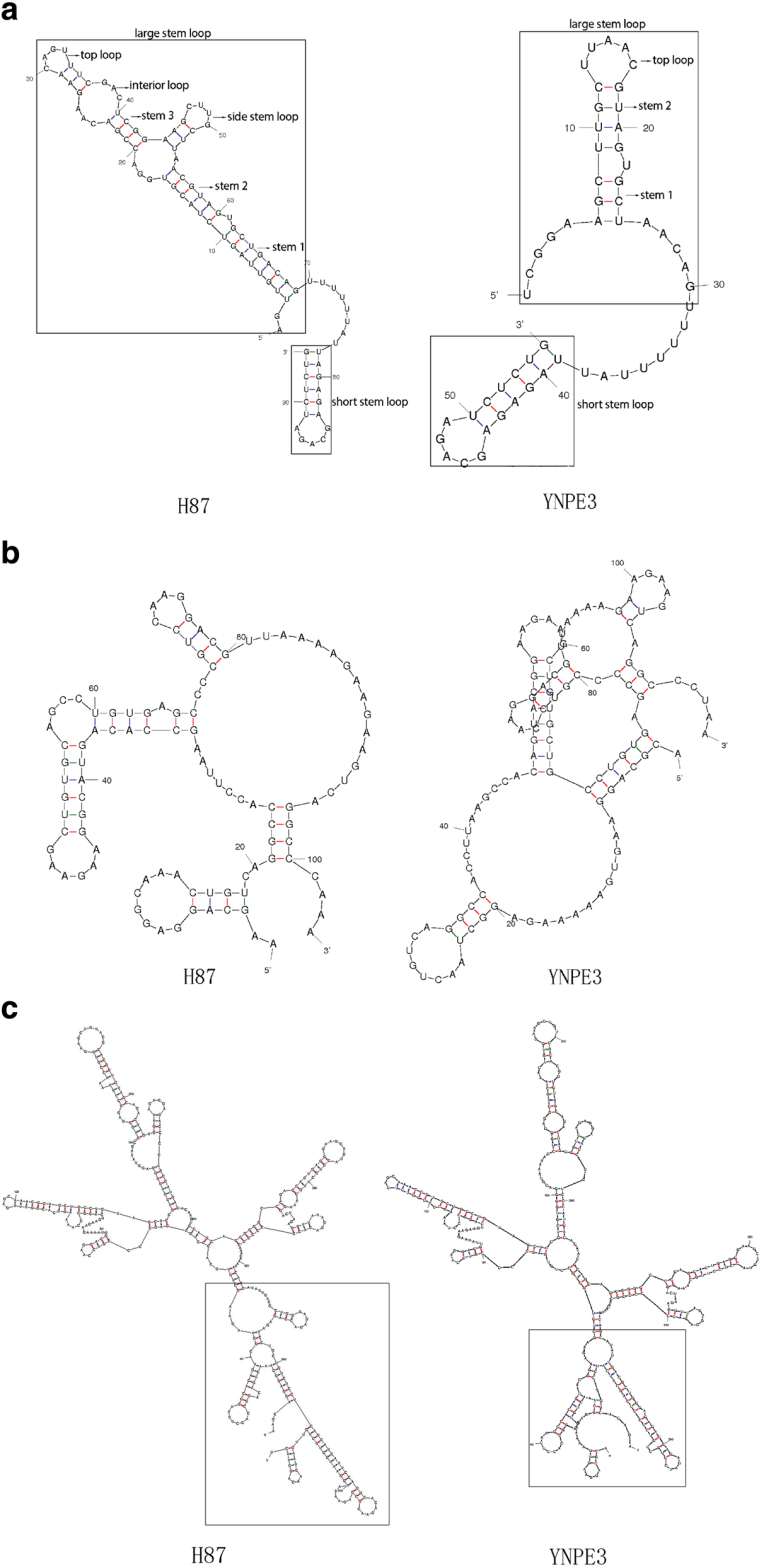
Prediction of secondary structure of 5′ UTR and 3′ UTR of strain YNPE3 and compared with the standard strain H87. **a** Prediction secondary structure of the whole 5′ UTR from the YNPE3 strain (nucleotides 1 to 55) and the prototype strain H87 (nucleotides 1 to 94). **b** Prediction secondary structure of the 3′ UTR variable region from the YNPE3 strain (nucleotides 1 to115) and strain H87 (nucleotides 1 to 104). **c** prediction secondary structure of the whole 3′ UTR from the prototype strain H87 and the YNPE3 strain

Moreover, two fragments of about 40 nts, including 1-AGTTGTTAGTCTACGTGGACCGACAAGAACAGTTTCGAC-39 for 5′ UTR and 389-ACGCCAGAAAATGGAATGGTG CTGTTGAATCAACAGGTTCT-429 for 3′ UTR were deleted from the YNPE3 strain, and 11 nts 9-ACGCAGGAAGT-19 insertion presented in the hyper-variable region of the 3′ UTR of the YNPE3 strain when compared with strain H87. In view of the important role of the 3′ and 5′ UTR of Flaviviruses in viral replication and pathogenesis [35, 36], we analyzed the RNA secondary structures of the 3′ and 5′ UTR of the YNPE3 strain by MFOLD software that was used to predict RNA secondary structures. The results showed that two stem-loops were observed in the 5′ UTR of the YNPE3 and strain H87 (Fig. 3a). However, due to the deletion of 39 nts in the 5′ UTR of the YNPE3 strain, the large stem loop showed a truncated secondary structure. In addition, the secondary structure in variable region (VR) of the 3′ UTR of the YNPE3 strain was significantly different from that of the prototype H87 strain, the strain YNPE3 presented a unique secondary structure (Fig. 3b). As show in Fig. 3c, the overall secondary structure of 3′ UTR of the YNPE3 strain was consistent with H87; only at the terminal part it presented a distinctive structure due to the nucleotides deletions and insertions.

### Amino acid substitution analysis of YNPE3

To determine the variations of the amino acids of the YNPE3 strain, the complete amino acid sequences of eight closely related reference strains were retrieved from GenBank database (http://www.ncbi.nlm.nih.gov). They were subjected to multiple sequence alignment analysis. As shown in Table 1, compared with strain H87, a total of 75 amino acid substitutions were found in the whole polyprotein of the YNPE3 strain with a substitution rate of 2.2%. These substitutions occurred mainly in the E, NS1, NS3 and NS5 proteins of YNPE3. However, the PrM/M protein was relatively conserved, and there were no amino acid substitutions found. Also, less amino acids substitutions were found in the capsid, NS2B and NS4A proteins of YNPE3. Compared with strain H87, many of the amino acid substitutions in the YNPE3 strain were conservative, and they mainly consisted of exchange of amino acids with similar physicochemical properties; Whereas, few amino acids mutations were involved in changes in polarity, such as E_169:_ A(Ala) → T(Thr), E_301:_ L(Leu) → T(Thr), NS1_93:_ I(Ile) → T(Thr) and NS5_188:_ T(Thr) → A(Ala). Moreover, the strain YNPE3 and Balotra87-s shared the same unique amino acid replacements in some sites.

**Table 1:** The major amino acid substitutions among the seven related strains of DENV-3 from China, Laos, India and Singapore as compared with the standard strain H87

Aa position		H87	YNPE3	Balotra87_s	YN01	UI17760	D3/SG/CT7/2012
		Philippines 1956	China 2013	India 2013	China 2013	Laos 2010	Singapore 2012
		GV	GIII	GIII	GII	GII	GI
ORF	Protein						
Capsid							
35	35	R	K	K			
86	86	K	R				
108	108	M	I	I			
Envelope							
361	81	I	V	V			T
404	124	S	L	P			L
412	132	H	Y	Y			
420	140	I			T	T	
434	154	E			D	D	
444	164	S	P	P	P	P	
449	169	A	T	T	V	V	P
505	225	K	E	E	E	E	V
550	270	T	N	N	N	N	E
551	271	T	S	S	S	S	T
572	292	K	E	E	E	E	S
581	301	L	T	T			E
663	383	K	N	N			S
671	391	R	K	K	K	K	
727	447	S			G	G	K
732	452	I	V	V			
NS1							
820	47	R	K				
821	48	V	L	L	L	L	L
856	83	D	N	N	N	N	N
866	93	I	T	T			
867	94	T	I	I	I	I	I
871	98	E	D				
893	120	L	K	K	K	K	K
912	139	S	N	N	N	N	N
951	178	L	M	M	T	T	L
961	188	V			I	I	
990	217	L			F	F	
1029	256	H	Y	Y			Y
1061	288	S	T	T	T	T	T
1112	339	N	S	S			
NS2A							
1162	37	L	F	F			
1225	100	E	K				
1237	112	A	T	T	T	T	T
1240	115	R	Q	Q	Q	Q	Q
1267	142	T	I				
1275	150	V	I	I			
1300	175	I	V	V	V	V	
NS3							
1504	31	F	L	L			
1588	115	I	T	T			
1829	356	V	A	A			
1865	392	D	E	E			
1910	437	D	E				
1925	452	V	A	A	A	A	
1949	476	M	T	T		T	T
1962	489	W	E				
1964	491	E	Q				
1965	492	A	E				
2041	568	E	Q	Q			
NS4A							
2162	70	L	I				
2191	99	D	E	E			
2192	100	V	I	I			I
2240	148	V	I	I			
NS4B							
2357	115	V	I	I			
2393	151	M	I	I			
NS5							
2540	50	I	T	T	T	T	
2678	188	T	A	A			A
2778	288	S	N	N			
2912	422	R	K	K			
3052	562	Q	L	L			
3075	585	K	T	T			
3109	619	I	V	V			
3129	639	L	P	P			
3130	640	E	G				A
3253	763	T	S	S	S	S	
3325	835	D	N	N			
3354	864	L	S	P			

The GI, GII, GIII and GV represent genotype I, genotype II, genotype III and genotype V, respectively. The accession number of those related strains: H87(M93130), Balotra87_s(KU216209), YN01(KF824902), UI17760(KY849769), D3/SG/CT7/2012(KX380839). YNPE3(MF370226)

### Phylogenetic analysis

**Fig. 4:**
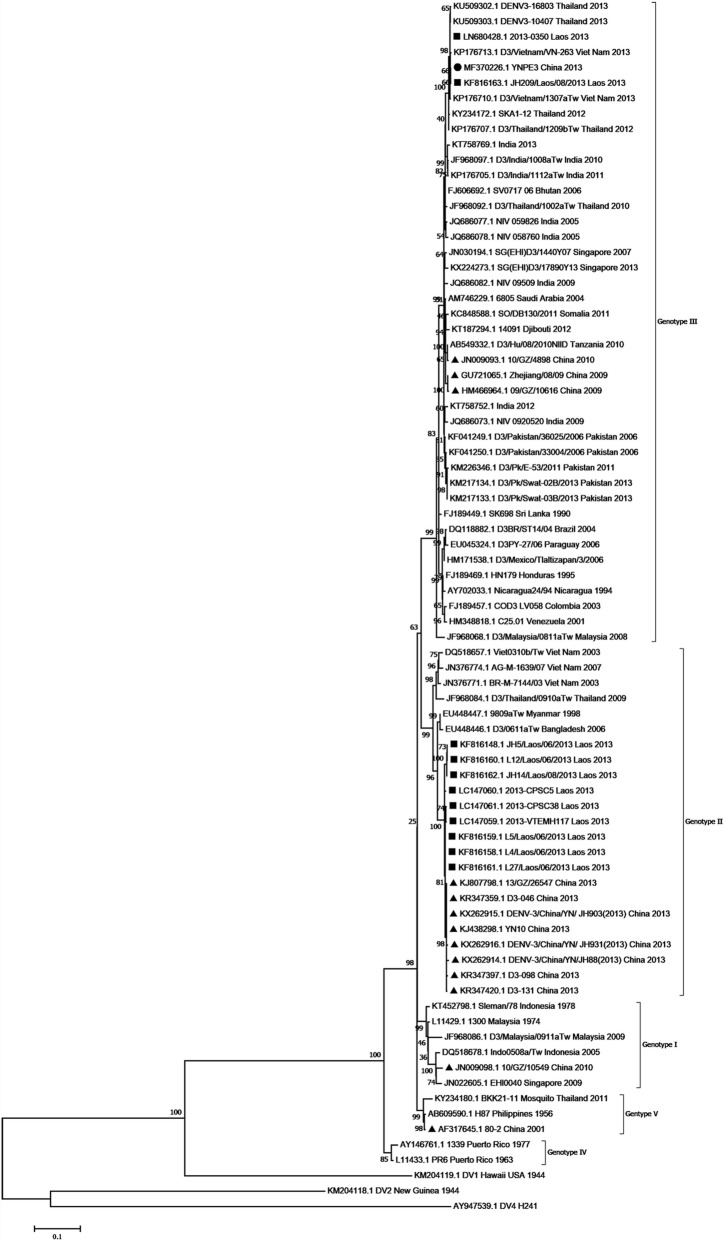
Phylogenetic tree based on envelope gene of DENV-3 strains obtained by Maximum-Likelihood method in MEGA7.0. Black dot represents DENV-3 sequence isolated in current study, the Laos and other Chinese DENV-3 isolates are marked with black squares and triangles, respectively. A total of 76 reference strains of the five genotypes available in NCBI GenBank were used for comparison. The percentage of replicate trees in which the associated taxa clustered together in the bootstrap test (1000 replicates) are shown next to the branches. The scale bar expresses the genetic distance. DENV strains are named as follows: GenBank accession number/strain/country/year

**Fig. 5:**
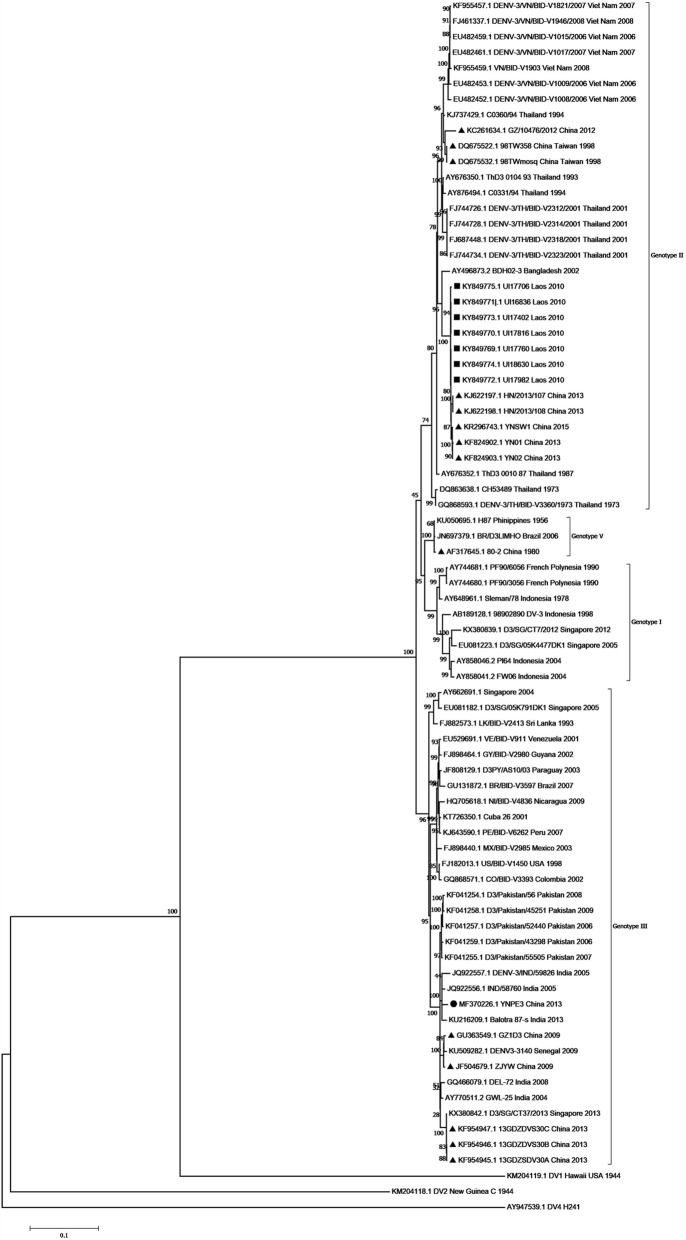
Phylogenetic tree based on the complete coding sequences of DENV-3 strains obtained by Maximum-Likelihood method in MEGA7.0. Black dot represents DENV-3 sequence isolated in current study. The Laos and other Chinese DENV-3 isolates are marked with black squares and triangles, respectively. A total of 75 reference strains of the four genotypes available in NCBI GenBank are retrieved. The percentage of replicate trees in which the associated taxa clustered together in the bootstrap test (1000 replicates) are shown next to the branches. The scale bar expresses the genetic distance. DENV strains are named as follows: GenBank accession number/strain/country/year

To determine the evolutionary history of the YNPE3 strain, two phylogenetic trees were drawn based on the E gene and the CDS of DENV-3. The phylogenetic tree based on the E gene revealed that DENV-3 strains had five distinct genotypic groups (Fig. 4). The YNPE3 strain was clustered into genotype III and was closely related to the contemporaneous DENV-3 strains from Laos, Vietnam, Thailand and India. Interestingly, seven Yunnan isolates (KR347359, KX262915, KJ438298, KR347397, KR347420, KX262916, KX262914), and nine strains from Laos (KF816148, KF816160, KF816162, LC147060, KF816158, KF816159, KF816161, LC147061, LC147059) that were identified in 2013, belonged to genotype II. Moreover, the phylogenetic tree based on CDS revealed that the YNPE3 strain was grouped into genotype III along with other DENV-3 isolates from different regions (Fig. 5). Phylogenetically, the strains Balotra87-s (KU216209), IND/58760 (JQ992556), DENV-3/IND/59826 (JQ992556) from India and two strains from China (JF504679, GU363549) were closely related to the YNPE3 strain. Interestingly, the YN01 and YN02 strains isolated in Yunnan in 2013 were classified into genotype II and were located in the same clades with seven strains isolated in Laos in 2010. Overall, the two trees suggested that the current strain belonged to the genotype III of DENV-3.

## Discussion

Over recent years, dengue has become a serious public health concern in China. The imported cases of dengue from other countries, and especially Southeast Asia countries, have caused a regional epidemic in Yunnan, China [26, 27, 37]. Therefore, disease surveillance and prevention employing genome sequencing and molecular and evolutionary studies of DENV isolates are of great importance. As stated in the introduction, dengue outbreak in Yunnan was related to the outbreak in Laos. In current research, the phylogenetic analysis based on E gene suggested that the YNPE3 strain was closely related to the contemporaneous strains isolated in Laos. Previous study has revealed a strong relationship between the Laos 2013 isolates genotype III and strains from Bhutan (2007) and India (2005) [22]. Especially, the phylogenetic tree based on E gene indicated that strains from Thailand, Laos, Vietnam and Pakistan had a strong relationship with the India isolates of genotype III (from 2005 to 2013). The phylogenetic tree based on CDS also supported that YNPE3 strain had a close relationship with India isolates in 2005 (strains IND/58760 and DENV-3/IND/59826) and 2013 (strain Balotra87-s). These strains shared 98.2–98.9% nucleotide identity with YNPE3 strain. Nevertheless, the precise evolutionary relationship between strain YNPE3 and strains from Laos and India could not be confirmed due to lack of complete coding sequences information on genotype III from Laos during 2005–2013. Yet, previous studies have reported the emergence of genotype III from 2009 to 2011 in India and China [38, 39]. This genotype has re-emerged in Southeast Asian countries and it has been in circulation for a very long time. Moreover, the two phylogenetic trees indicated genotype II isolated in Yunnan was closely related with Laos strains isolated in 2010 and 2013, which suggested that this genotype most likely had common ancestor with the genotype II strains circulating in Laos.

Previous studies have indicated an important role of the secondary structure of the 5′ and 3′ UTR of the *Flaviviruses* in viral replication [40, 41], The deletion and insertion of bases in the 5′ and 3′ UTR probably leads to changes in RNA secondary structures. For most DENV isolates, the 3′ UTR of the genome contains three functional domains [40, 42]. The nucleotide insertions and mutations usually occur in domain I, which is close to the NS5 protein and is regarded as the variable region of the 3′ UTR of DENV-3 [35, 42], The potential effect of the insertion and mutation has shown to be related with viral replication efficiency [36, 43, 44]. Our results revealed that the insertion of 11 nts occurred in domain I of the 3′ UTR of the YNPE3 strain, while the secondary structure of VR was different from that of strain H87, which was consistent with the results of a previous study [45]. However, no significant change occurred in the whole secondary structure of 3′ UTR. The 5′ UTR has two domains, including the short stem loop domain II and large stem loop domain I, which are separated by a functional spacer oligo U sequence. Domain I is the promoter of the viral RNA-dependent RNA polymerase (RdRp). It comprises several conserved structures: three helix regions (Stem 1, Stem 2 and Stem 3), a top loop and a side stem loop [35, 46]. Most of the variations in sequence and structure occur in the side loop and Stem 3 helix region. The replication of the virus is affected if the side loop is deleted [35, 46]. Interestingly, the side loop and Stem 3 helix region of the YNPE3 strain were deleted. We further compared the differences in secondary structure of 5′ UTR and 3′ UTR between Yunnan strains YN01, YN02 and our strain YNPE3. The YN01 and YN02 revealed a same structure with H87 in 5′ UTR but a distinctive structure in 3′ UTR (data not show). Due to lack of a complete genome sequence of DENV-3 from Laos, no comparison could be made between YNPE3 and strains from Laos. Hence, further investigation is needed to elucidate the biological implication of these deletions and insertions.

As major viral antigen, the E protein can be recognized by host cells, thus inducing neutralizing antibodies and blocking membrane fusion and virion assembly [47–49]. Moreover, the virulence of dengue virus mainly depends on the E region. The mutations of amino acids in conserved regions of the E protein affect the viral attachment in host cells [47]. The E_383:_ K(Lys) → N(Asn) and E_391_R(Arg) → K(Lys) might be related to the severity of the disease of DENV-3 [50]. The mutations that occur in the E_124:_ S(Ser) → P(Pro), E_169:_ A(Ala) → T(Thr) and E_383:_ K(Lys) → N(Asn) are usually characteristics of genotype III [51]. The E_169:_ A(Ala) → T(Thr) and E_301:_ L(Leu) → T(Thr) are two important non-conservative replacements that involve the nonpolar hydrophobic amino acid to polar amino acid. The E-169, as a positively selected site [52], has been shown to be located in a murine B and T cell epitope [53]. These mutations might be related to the transmission capacity of these isolates [54]. Similar substitutions referring to shifts of polarity of amino acids were observed in other proteins, particularly in non-structural proteins NS1, NS2A, NS3 and NS5. Importantly, further studies are needed to confirm the actual functions of these substitutions. Like genotype III, genotype II strains from Laos and China also have exclusive amino acid substitution characteristics. We concluded that the same genotype, or the highly related strains shared some common characteristics in amino acid substitutions.

## Conclusions

In conclusion, a full-length genome of an imported DENV-3 strain YNPE3 from Laos was reported for the first time. Deletions of nucleotides in the 3′ and 5′ UTR were observed. Studying the amino acid substitutions can lead to a better understanding of viral pathogenesis, which in turn might promote the development of new DENV vaccine. The genotype III and genotype II of DENV-3 from Yunnan were strongly related to those DENV-3 strains from Southeast Asia countries. Thus, the dengue surveillance and warning systems in Yunnan province of China need to be strengthened by timely monitoring of the two DENV-3 genotypes, that are prevalent in Southeast Asia.

## Additional files

Additional file 1: **Table S1.** Typical primers of dengue virus. (DOC 32 kb)

Additional file 2: **Table S2.** Primers used for the complete genome amplification. (DOC 38 kb)

Additional file 3: **Table S3.** Sequence information used in E gene phylogenetic tree construction. (DOC 88 kb)

Additional file 4: **Table S4.** Sequence information used in the complete coding sequence phylogenetic tree construction. (DOC 88 kb)

## Abbreviations

Aa: Amino acids
BLAST: Basic local alignment search tool
C: Capsid protein
CDS: Complete coding sequence
CPE: Cytopathic effect
DENV: Dengue virus
DENV-3: Dengue virus serotype 3
DF: Dengue fever
DHF: Dengue hemorrhagic fever
DSS: Dengue shock syndrome
E: Envelope glycoprotein
GI: Genotype I
GII: Genotype II
GIII: Genotype III
GV: Genotype V
M: Membrane glycoprotein
NS: Nonstructural proteins
nts: nucleotides
ORF: Open reading frame
PrM: Membrane glycoprotein precursor
RdRp: RNA-dependent RNA polymerase
RPMI: Roswell Park Memorial Institute
RT-PCR: Reverse transcriptase polymerase chain reaction
UTR: Untranslated region
VR: Variable region
