# Supplementary material for: Molecular characterization and phylogenetic analysis of a dengue virus serotype 3 isolated from a Chinese traveler returned from Laos
Source: Virol J. 2018 Jul 24;15:113. doi: 10.1186/s12985-018-1016-5 (PMC6057004; doi:10.1186/s12985-018-1016-5)
Supplement: Supplementary file 1 — Table S1. Typical primers of dengue virus. (DOC 32 kb) [file 12985_2018_1016_MOESM1_ESM.doc]

**Table S1 Typical primers of dengue virus**

| Primer name | sequence（5′→3′） | type specificity | Product (bp) |
| --- | --- | --- | --- |
| D1 | TCAATATGCTGAAACGCGCGAGAAACCG | DENV | 511 (D1 and D2) |
| D2 | TTGCACCAACAGTCAATGTCTTCAGGTTC | DENV |
| TS1 | CGTCTCAGTGATCCGGGGG | DENV-1 | 482 (D1 and TS1) |
| TS2 | CGCCACAAGGGCCATGAACAG | DENV-2 | 119 (D1 and TS2) |
| TS3 | TAACATCATCATGAGACAGAGC | DENV-3 | 290 (D1 and TS3) |
| TS4 | CTCTGTTGTCTTAAACAAGAGA | DENV-4 | 392 (D1 and TS4) |
