# Supplementary material for: Molecular characterization and phylogenetic analysis of a dengue virus serotype 3 isolated from a Chinese traveler returned from Laos
Source: Virol J. 2018 Jul 24;15:113. doi: 10.1186/s12985-018-1016-5 (PMC6057004; doi:10.1186/s12985-018-1016-5)
Supplement: Supplementary file 2 — Table S2. Primers used for the complete genome amplification. (DOC 38 kb) [file 12985_2018_1016_MOESM2_ESM.doc]

**Table S2 Primers used for the** complete genome amplification.

| Primer name | sequence（5′→3′） | positions | Product (bp) |
| --- | --- | --- | --- |
| 3F1 | AGTTGTTAGTCTACGTGGACCG | 1-22 | 1424 |
| 3R1 | GTGTTATCTCAACCGTGACTCC | 1403-1424 |
| 3F2 | GGAGCAGGACCAAAACTACG | 1186-1205 | 1421 |
| 3R2 | TCTCCATTCTGGTTGTTGACC | 2586-2606 |
| 3F3 | ACACTCTATCTGGGAGCTGTGG | 2384-2405 | 1436 |
| 3R3 | GGCAGTTGTAACGTTGTTGC | 3800-3819 |
| 3F4 | GTTTGTGCTCCTTCTCTCAGG | 3586-3606 | 1426 |
| 3R4 | CGCTATTCCACTGACGTAGC | 4992-5011 |
| 3F5 | AGGTGCAGGTTATTGCCGTAG | 4794-4814 | 1427 |
| 3R5 | ACGTTCTCCATCAAAGCACC | 6201-6220 |
| 3F6 | GGCCAGCCTCTCAACAATG | 5942-5960 | 1494 |
| 3R6 | CCAGAGTGTTGTTATTGGTCC | 7415-7435 |
| 3F7 | CCAGGATTGCAGGCTAAAGC | 7187-7206 | 1417 |
| 3R7 | GAGTTGTGTCTGTCATTGCC | 8584-8603 |
| 3F8 | ACATGTCAATGCGGAACCAG | 8381-8400 | 1460 |
| 3R8 | GACTCCACATTTGGGCGTAG | 9821-9840 |
| 3F9 | ACAGGTTCGCTAATGCCCTG | 9579-9598 | 1118 |
| 3R9 | AGAACCTGTTGATTCAACAGCAC | 10675-10696 |

F: upstream primer, R: downstream primer
