# Supplementary material for: Molecular characterization and phylogenetic analysis of a dengue virus serotype 3 isolated from a Chinese traveler returned from Laos
Source: Virol J. 2018 Jul 24;15:113. doi: 10.1186/s12985-018-1016-5 (PMC6057004; doi:10.1186/s12985-018-1016-5)
Supplement: Supplementary file 3 — Table S3. Sequence information used in E gene phylogenetic tree construction. (DOC 88 kb) [file 12985_2018_1016_MOESM3_ESM.doc]

**Table S3** Sequence information used in E gene phylogenetic tree construction

| **Accession** | **Strain** | **Location** | **Year** |
| --- | --- | --- | --- |
| LN680428 | 2013-0350 | Laos | 2013 |
| LC147061 | 2013-CPSC38 | Laos | 2013 |
| LC147060 | 2013-CPSC5 | Laos | 2013 |
| LC147059 | 2013-VTEMH117 | Laos | 2013 |
| KF816162 | JH14/Laos/08/2013 | Laos | 2013 |
| KF816163 | JH209/Laos/08/2013 | Laos | 2013 |
| KF816148 | JH5/Laos/06/2013 | Laos | 2013 |
| KF816160 | L12/Laos/06/2013 | Laos | 2013 |
| KF816161 | L27/Laos/06/2013 | Laos | 2013 |
| KF816158 | L4/Laos/06/2013 | Laos | 2013 |
| KF816159 | L5/Laos/06/2013 | Laos | 2013 |
| KY234180 | BKK21-11 Mosquito | Thailand | 2011 |
| JF968084 | D3/Thailand/0910aTw | Thailand | 2009 |
| JF968092 | D3/Thailand/1002aTw | Thailand | 2010 |
| KP176707 | D3/Thailand/1209bTw | Thailand | 2012 |
| KU509303 | DENV3-10407 | Thailand | 2013 |
| KY234172 | SKA1-12 | Thailand | 2012 |
| KU509302 | DENV3-16803 | Thailand | 2013 |
| JQ686077 | NIV_059826 | India | 2005 |
| JQ686082 | NIV_09509 | India | 2009 |
| JF968097 | D3/India/1008aTw | India | 2010 |
| KP176705 | D3/India/1112aTw | India | 2011 |
| JQ686078 | NIV 058760 | India | 2005 |
| JQ686073 | NIV 0920520 | India | 2009 |
| KT758752 |  | India | 2012 |
| KT758769 |  | India | 2013 |
| AB549332 | D3/Hu/Tanzania/08/2010NIID | Tanzania | 2010 |
| FJ606692 | SV0717_06 | Bhutan | 2006 |
| AM746229 | 6805 | Saudi Arabia | 2004 |
| KC848588 | SO/DB130/2011 | Somalia | 2011 |
| KF041249 | D3/Pakistan/36025 | Pakistan | 2006 |
| KF041250 | D3/Pakistan/33004/2006 | Pakistan | 2006 |
| KM226346 | D3/Pk/E-53/2011 | Pakistan | 2011 |
| KM217134 | D3/Pk/Swat-02B/2013 | Pakistan | 2013 |
| KM217133 | D3/Pk/Swat-03B/2013 | Pakistan | 2013 |
| EU448447 | 9809aTW | Myanmar | 1998 |
| L11429 | 1300 | Malaysia | 1974 |
| JF968068 | D3/Malaysia/0811aTw | Malaysia | 2008 |
| JF968086 | D3/Malaysia/0911aTw | Malaysia | 2009 |
| KT187294 | Djibouti_2012_14091 | Djibouti | 2012 |
| GU721065 | Zhejiang/08/09 | China | 2009 |
| AF317645 | 80-2 | China | 2001 |
| KR347359 | D3-046 | China | 2013 |
| KR347397 | D3-098 | China | 2013 |
| KR347420 | D3-131 | China | 2013 |
| HM466964 | 09/GZ/10616 | China | 2009 |
| JN009098 | 10/GZ/10549 | China | 2010 |
| JN009093 | 10/GZ/4898 | China | 2010 |
| KJ807798 | 13/GZ/26547 | China | 2013 |
| KX262915 | DENV-3/China/YN/ JH903(2013) | China | 2013 |
| KX262916 | DENV-3/China/YN/ JH931(2013) | China | 2013 |
| KX262914 | DENV-3/China/YN/JH88(2013) | China | 2013 |
| KJ438298 | YN10 | China | 2013 |
| KT452798 | Sleman/78 | Indonesia | 1978 |
| DQ518678 | Indo0508a/TW | Indonesia | 2005 |
| FJ189449 | SK698 | Sri Lanka | 1990 |
| FJ189469 | HN179 | Honduras | 1995 |
| HM171538 | D3/Mexico/Alto Lucero/5/2006 | Mexico | 2006 |
| FJ189457 | COD3_LV058 | Colombia | 2003 |
| DQ118882 | D3BR/ST14/04 | Brazil | 2004 |
| HM348818 | C25.01 | Venezuela | 2001 |
| AB609590 | H87 | Philippines | 1956 |
| EU448446 | D3/Bangladesh/0611aTw | Bangladesh | 2006 |
| DQ518657 | Viet0310b/Tw | Viet Nam | 2003 |
| JN376774 | AG-M-1639/07 | Viet Nam | 2007 |
| JN376771 | BR-M-7144/03 | Viet Nam | 2003 |
| KP176710 | D3/Vietnam/1307aTw | Viet Nam | 2013 |
| KP176713 | D3/Vietnam/VN-263 | Viet Nam | 2013 |
| JN022605 | EHI0040 | Singapore | 2009 |
| JN030194 | SG(EHI)D3/1440Y07 | Singapore | 2007 |
| KX224273 | SG(EHI)D3/17890Y13 | Singapore | 2013 |
| AY702033 | Nicaragua24/94 | Nicaragua | 1994 |
| AY146761 | 1339 | Puerto Rico | 1977 |
| L11433 | PR6 | Puerto Rico | 1963 |
| EU045324 | D3PY-27/06 | Paraguay | 2006 |
| KM204119 | Hawaii | USA | 1944 |
| KM204118 | New Guinea | New Guinea | 1944 |
| AY947539 | H241 | NA | NA |
| MF370226 | YNPE3 | China | 2013 |
