# Supplementary material for: Molecular characterization and phylogenetic analysis of a dengue virus serotype 3 isolated from a Chinese traveler returned from Laos
Source: Virol J. 2018 Jul 24;15:113. doi: 10.1186/s12985-018-1016-5 (PMC6057004; doi:10.1186/s12985-018-1016-5)
Supplement: Supplementary file 4 — Table S4. Sequence information used in the complete coding sequence phylogenetic tree construction. (DOC 88 kb) [file 12985_2018_1016_MOESM4_ESM.doc]

**Table S4: Sequence information used in the whole genome phylogenetic tree construction**

| **Accession** | **strain** | **location** | **year** |
| --- | --- | --- | --- |
| KF954946 | 13GDZDVS30B | China | 2013 |
| KF954947 | 13GDZDVS30C | China | 2013 |
| KF954945 | 13GDZSDV30A | China | 2013 |
| KC261634 | GZ/10476/2012 | China | 2012 |
| KJ622197 | HN/2013/107 | China | 2013 |
| KJ622198 | HN/2013/108 | China | 2013 |
| KF824902 | YN01 | China | 2013 |
| KF824903 | YN02 | China | 2013 |
| JF504679 | ZJYW | China | 2009 |
| GU363549 | GZ1D3 | China | 2009 |
| DQ675522 | 98TW358 | China | 1998 |
| DQ675532 | 98TWmosq | China | 1998 |
| KR296743 | YNSW1 | China | 2015 |
| AF317645 | 80-2 | China | 1980 |
| KY849771 | UI16836 | Laos | 2010 |
| KY849773 | UI17402 | Laos | 2010 |
| KY849775 | UI17706 | Laos | 2010 |
| KY849769 | UI17760 | Laos | 2010 |
| KY849770 | UI17816 | Laos | 2010 |
| KY849772 | UI17982 | Laos | 2010 |
| KY849774 | UI18630 | Laos | 2010 |
| KJ737429 | C0360/94 | Thailand | 1994 |
| DQ863638 | CH53489 | Thailand | 1973 |
| FJ744726 | DENV-3/TH/BID-V2312/2001 | Thailand | 2001 |
| FJ744728 | DENV-3/TH/BID-V2314/2001 | Thailand | 2001 |
| FJ687448 | DENV-3/TH/BID-V2318/2001 | Thailand | 2001 |
| FJ744734 | DENV-3/TH/BID-V2323/2001 | Thailand | 2001 |
| GQ868593 | DENV-3/TH/BID-V3360/1973 | Thailand | 1973 |
| AY676352 | ThD3_0010_87 | Thailand | 1987 |
| AY676350 | ThD3 0104 93 | Thailand | 1993 |
| AY876494 | C0331/94 | Thailand | 1994 |
| EU482452 | DENV-3/VN/BID-V1008/2006 | Viet Nam | 2006 |
| EU482453 | DENV-3/VN/BID-V1009/2006 | Viet Nam | 2006 |
| EU482459 | DENV-3/VN/BID-V1015/2006 | Viet Nam | 2006 |
| EU482461 | DENV-3/VN/BID-V1017/2007 | Viet Nam | 2007 |
| KF955457 | DENV-3/VN/BID-V1821/2007 | Viet Nam | 2007 |
| FJ461337 | DENV-3/VN/BID-V1946/2008 | Viet Nam | 2008 |
| KF955459 | VN/BID-V1903 | Viet Nam | 2008 |
| KF041258 | D3/Pakistan/45251 | Pakistan | 2009 |
| KF041259 | D3/Pakistan/43298 | Pakistan | 2006 |
| KF041257 | D3/Pakistan/52440 | Pakistan | 2006 |
| KF041255 | D3/Pakistan/55505 | Pakistan | 2007 |
| KF041254 | D3/Pakistan/56 | Pakistan | 2008 |
| KX380842 | D3/SG/CT37/2013 | Singapore | 2013 |
| KX380839 | D3/SG/CT7/2012 | Singapore | 2012 |
| AY662691 | NA | Singapore | 2004 |
| EU081182 | D3/SG/05K791DK1 | Singapore | 2005 |
| EU081223 | D3/SG/05K4477DK1 | Singapore | 2005 |
| GQ466079 | DEL-72 | India | 2008 |
| JQ922557 | DENV-3/IND/59826 | India | 2005 |
| JQ922556 | IND/58760 | India | 2005 |
| KU216209 | Balotra 87-s | India | 2013 |
| AY770511.2 | GWL-25 | India | 2004 |
| KU509282 | DENV3-3140 | Senegal | 2009 |
| FJ182013 | US/BID-V1450 | USA | 1998 |
| EU52969 | VE/BID-V911 | Venezuela | 2001 |
| KT726350 | Cuba_26 | Cuba | 2001 |
| FJ882573 | LK/BID-V2413 | Sri Lanka | 1993 |
| KJ643590 | PE/BID-V6262 | Peru | 2007 |
| FJ898464 | GY/BID-V2980 | Guyana | 2002 |
| GQ868571 | CO/BID-V3393 | Colombia | 2002 |
| JF808129 | D3PY/AS10/03 | Paraguay | 2003 |
| FJ898440 | MX/BID-V2985 | Mexico | 2003 |
| HQ705618 | NI/BID-V4836 | Nicaragua | 2009 |
| GU131872 | BR/BID-V3597 | Brazil | 2007 |
| JN697379 | BR/D3LIMHO | Brazil | 2006 |
| KU050695 | H87 | Phinippines | 1956 |
| AY648961 | Sleman/78 | Indonesia | 1998 |
| AB189128 | 98902890DV-3 | Indonesia | 1998 |
| AY858046.2 | PI64 | Indonesia | 2004 |
| AY858041.2 | FW06 | Indonesia | 2004 |
| AY744681 | PF90/6056 | French Polynesia | 1990 |
| AY744680 | PF90/3056 | French Polynesia | 1990 |
| AY496873.2 | BDH02-3 | Bangladesh | 2002 |
| KM204119 | Hawaii | USA | 1944 |
| KM204118 | New Guinea C | New Guinea | 1944 |
| AY947539  MF370226 | H241  YNPE3 | Singapore  China | NA  2013 |

NA: unclear.
